# Myocardial extracellular volume estimation by CMR predicts functional recovery following acute myocardial infarction

**DOI:** 10.1186/1532-429X-17-S1-Q63

**Published:** 2015-02-03

**Authors:** Ananth Kidambi, Manish Motwani, Akhlaque Uddin, David P Ripley, Adam K McDiarmid, Peter P Swoboda, David A Broadbent, Tarique A Musa, Bara Erhayiem, Josh Leader, John P Greenwood, Sven Plein

**Affiliations:** 1 Multidisciplinary Cardiovascular Research Centre & Leeds Institute for Cardiovascular and Metabolic Medicine, Leeds University, Leeds, UK; 2 Division of Medical Physics & Multidisciplinary Cardiovascular Research Centre, Leeds Institute of Cardiovascular and Metabolic Medicine, University of Leeds, Leeds, UK

## Background

The transmural extent of myocardial infarction (MI) as assessed by late gadolinium enhancement (LGE) CMR predicts functional recovery. In acute MI (AMI), myocardial edema and effects of reperfusion therapy reduce the predictive accuracy of LGE. LGE assesses tissue dichotomously as "viable" or "non-viable", but does not consider the severity of tissue damage within the hyperenhanced infarct zone. Extracellular volume (ECV) estimation, using native and post-contrast T1 mapping CMR, allows for quantitative assessment of severity of myocardial damage. We aimed to assess if CMR-derived measurement of infarct ECV offers additional predictive value over LGE extent for contractile recovery in reperfused AMI.

## Methods

35 patients presenting with first ST-segment elevation AMI treated by primary percutaneous coronary intervention underwent acute (day 2) and convalescent (3 months) CMR at 3.0 Tesla. Cine imaging, tissue tagging, modified Look-Locker inversion T1 mapping (3-3-5 acquisition with 3x R-R interval recovery epochs) natively and 15 minutes post gadolinium-contrast administration and LGE imaging at 20 minutes were performed. The ability of acute infarct ECV and acute transmural extent of LGE to predict convalescent wall motion, ejection fraction (EF) and strain were compared. A per-patient analysis was performed using a region of interest corresponding to the core of the infarct, excluding any microvascular obstruction (MO). Segmental analysis was also performed and evaluated using a multilevel linear mixed-effects model to account for non-independence of segmental data.

## Results

**Figure 1:**
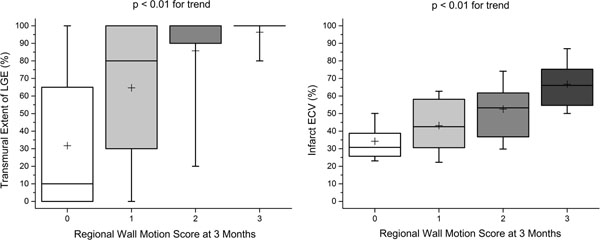
Comparison of convalescent regional wall motion score with LGE transmural extent (left panel) and infarct ECV (right panel). Note relatively wide ranges for transmural extent. Box denotes median, 25th and 75th percentiles, mean indicated by + and whiskers at 9th and 91st percentiles.

**Figure 2:**
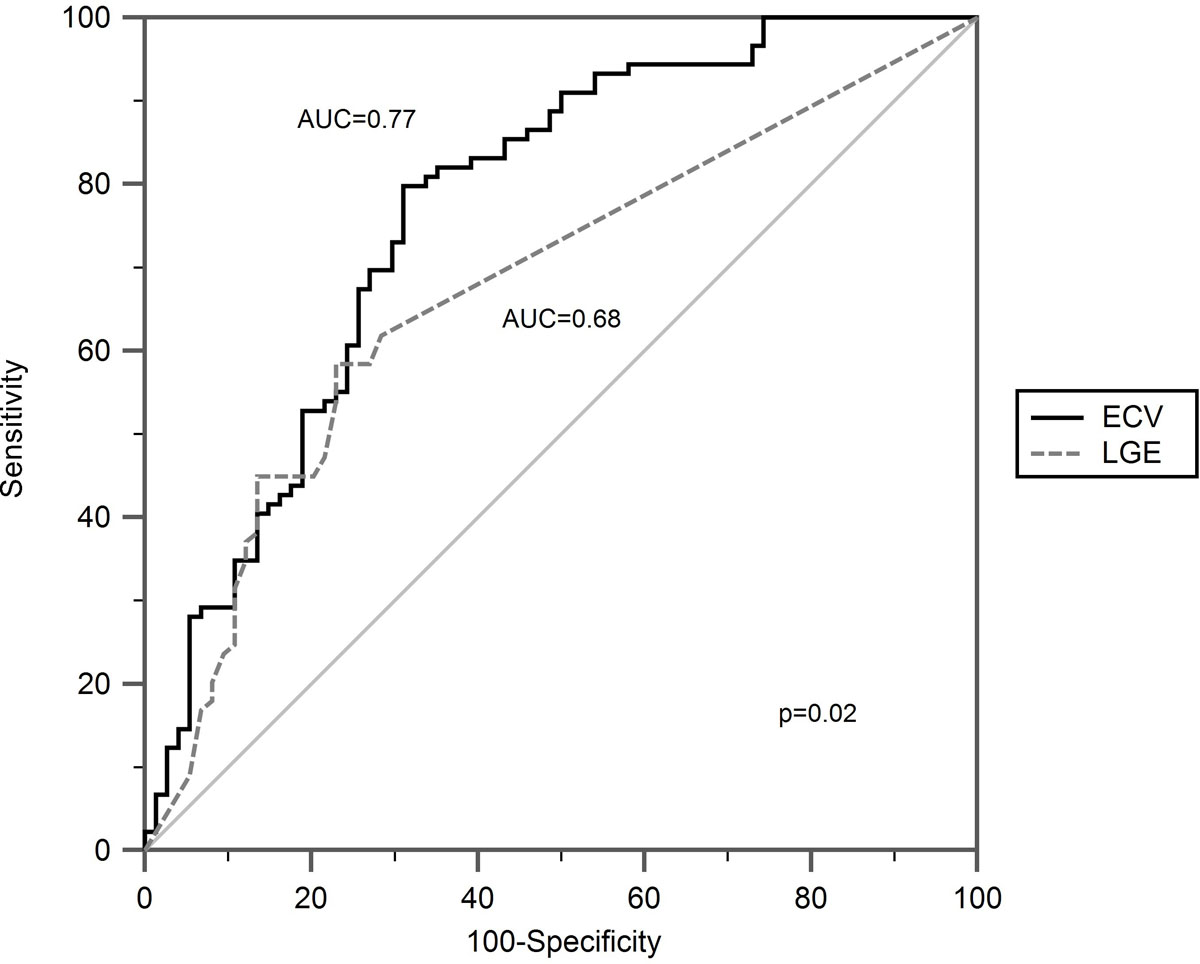
ROC curve comparing infarct ECV and transmural extent of infarction in dysfunctional segments (n=163) with improvement in wall motion score at 90 days. Remote segments not shown.

Per-patient, ECV and transmural extent of LGE correlated with convalescent wall motion score (r=0.43, p<0.01; r=0.41, p=0.02 respectively) and convalescent EF (r=-0.56, p<0.01; r=-0.36, p=0.04). Per-segment, acute ECV and LGE transmural extent were associated with convalescent wall motion score (β=0.55, r=0.54, p<0.01; β=0.50, r=0.50, p<0.01, Figure 1). ECV had higher accuracy than LGE extent to predict improvement in wall motion (area under receiver-operator-characteristics curve 0.77 vs. 0.68, p=0.02, Figure 2). ECV of ≤0.5 had sensitivity 81% and specificity 65% for prediction of improvement in segmental function. Adding ECV analysis to a 50% LGE transmural extent cut-off for prediction of improved wall motion in dysfunctional segments increased sensitivity from 87% to 89% and specificity from 34% to 81%. In multivariable analysis, acute infarct ECV was independently associated with both convalescent infarct strain and EF (β=0.58, p<0.001; β=-0.39, p=0.02) whereas LGE was not (β=0.13, p=0.35; β=-0.20, p=0.2). Acute infarct ECV in patients with and without MO was similar (0.54±0.18 vs. 0.57±0.10, p=0.6).

## Conclusions

Acute infarct ECV in reperfused AMI predicts regional and global LV remodeling, independent of transmural extent of infarction. Acute infarct ECV predicts functional recovery better than transmural infarct extent by LGE, demonstrating potential clinical utility for ECV mapping post-AMI.

## Funding

S.P is funded by British Heart Foundation fellowship (FS/10/62/28409).

